# The Correlation of Psychological Symptoms, Quality of Life and Resilience in Patients with Recurrent Non-Melanoma Skin Cancer

**DOI:** 10.3390/healthcare14172833

**Published:** 2026-09-03

**Authors:** Goran Šimić, Tomislav Sušac, Josip Lesko, Ana Dugandžić Šimić, Vedran Markotić, Dragan Babić

**Affiliations:** 1 School of Medicine, University of Mostar, 88000 Mostar, Bosnia and Herzegovina; tomislavsusac@gmail.com (T.S.); josip.lesko@mef.sum.ba (J.L.); ana.d.simic@gmail.com (A.D.Š.); vedranmarkoticz@gmail.com (V.M.); dragan.babic@tel.net.ba (D.B.); 2 Clinic for Otorhinolaryngology and Maxillofacial Surgery, University Clinical Hospital Mostar, 88000 Mostar, Bosnia and Herzegovina; 3 Clinic for Gynecology and Obstetrics, University Clinical Hospital Mostar, 88000 Mostar, Bosnia and Herzegovina; 4 Radiology Department, University Clinical Hospital Mostar, 88000 Mostar, Bosnia and Herzegovina

**Keywords:** non-melanoma skin cancer, psychological symptoms, quality of life, resilience, recurrent skin cancer

## Abstract

Recurrent non-melanoma skin cancer (NMSC) may be accompanied by cosmetic, functional, and emotional consequences. **Aims**: To examine associations among psychological symptoms, quality of life, resilience, and religiosity and to compare psychological outcomes between patients treated once and patients with recurrent facial NMSC. **Methods**: This cross-sectional study included 160 patients after surgery for facial NMSC (63 treated once and 97 with recurrent disease). Data were collected with a sociodemographic questionnaire, the Symptom Checklist-90-R (SCL-90-R), the Connor–Davidson Resilience Scale 25 (CD-RISC-25), the World Health Organization Quality of Life Scale (WHOQOL-BREF) and DUREL. **Results**: Greater psychological symptom severity was consistently associated with poorer scores across all WHOQOL-BREF domains. The overall multivariate group effect was significant (Pillai’s Trace = 0.235, *F*(6.149) = 7.641, *p* < 0.001, partial eta squared = 0.235). After Bonferroni correction for six follow-up tests, patients with recurrent NMSC had a higher adjusted GSI and lower adjusted psychological health scores than patients treated once (both *p* < 0.001). Adjusted differences in physical health, social relationships, environment, and CD-RISC were not statistically significant. **Conclusions**: Recurrent NMSC status was associated with greater psychological symptom burden and poorer psychological quality of life. Routine psychological screening may help identify patients who require further assessment or support.

## 1. Background

Non-melanoma skin cancer (NMSC) is the most common malignancy in humans. The incidence of NMSC has increased in recent years, affecting a younger population. Although the mortality rate is low, the treatment of NMSC is associated with high morbidity [1,2]. Approximately 80% of NMSCs occur on the head or neck, and it is projected that 40% of individuals who are diagnosed with their initial NMSC will also experience at least one more tumor within two years of their diagnosis [3]. NMSC is therefore considered to be a chronic, mutilating disease with great potential to leave cosmetic, functional, and emotional consequences.

For numerous cancer patients, receiving a diagnosis of cancer and going through treatment is a highly stressful situation that can lead to enduring negative psychological effects, such as emotional turmoil, depression, anxiety, sleep disturbances, fatigue, and a diminished quality of life [4,5,6,7,8]. Cancer is commonly perceived as a life-threatening and potentially traumatic illness, especially for its uncontrollable nature [9]. Furthermore, cancer patients must deal with dramatic life changes to which they have to adapt throughout their treatment process [10]. Studies released in the past few decades have revealed that even though cancer patients experience psychological and emotional issues, and the disease can significantly affect their quality of life if not addressed, many of these patients still do not receive specialized psychological or psychiatric care [11,12,13].

Advances in medical technology have improved the survivorship rate of cancer patients, but the issue of QOL during therapy and recovery has attracted widespread attention [14,15]. QOL is defined as an individual’s experience of his or her own state of being in terms of the goals, hopes, standards, and concerns that he or she pursues in the context of different value systems and cultures [16]. The quality of life (QOL) for cancer patients is a key measure of the success of clinical treatments and serves as a forecast for patients’ long-term survival [17]. Hence, evaluating and taking action to improve the quality of life of cancer patients is crucial. Various factors impact the quality of life of individuals with cancer, with psychological resilience being particularly significant [18].

Psychological resilience has been broadly defined as the ability to adapt to stressors, recover from adversities and life crises, and resume normal psychosocial functioning soon after adverse events [19]. Resilience’s protective role against distress in cancer patients can be influenced by various factors like positive feelings, mental adaptability (e.g., acceptance), proactive coping strategies, and religiosity, among others [10,20,21,22,23].

Due to stress caused by repeated surgical treatment and cancer diagnosis, we hypothesized that for subjects operated on for recurring non-melanoma skin cancers, the incidence and frequency of psychological symptoms would be reported higher than in subjects operated on for the same disease only once. Our aim was to examine associations among psychological symptoms, quality of life, resilience, and religiosity and to compare psychological outcomes between patients treated once and patients with recurrent facial NMSC.

## 2. Participants and Methods

A cross-sectional study was conducted at the Clinic for Otorhinolaryngology and Maxillofacial Surgery, University Clinical Hospital Mostar, from February 2025 to April 2026. The sample consisted of 160 adults aged 40–80 years who underwent surgery for facial NMSC and voluntarily agreed to participate. Questionnaires were completed at the first follow-up visit, within seven days after participants had been informed of the histopathological diagnosis and within three weeks after surgery. Participants were instructed to answer with reference to the period after receiving the histopathological diagnosis. Before questionnaire administration, they were informed that the tumor had been completely removed and that the planned surgical treatment had been completed. All participants provided written informed consent.

The recurrent NMSC group comprised 97 patients, and the treated-once group comprised 63 patients. Tumors were located on the face or neck and were up to 2 cm in diameter. Recurrent basal cell carcinoma or squamous cell carcinoma occurred between 4–6 months and three years after the previous operation and was confirmed by histopathological analysis. Patients in the recurrent group had been followed at the study clinic since their previous operation. No postoperative mutilation or facial dysfunction was recorded. The groups were not individually matched; their unequal sizes reflected the numbers of participants meeting the respective group definitions during the study period.

Exclusion criteria were a previous psychiatric diagnosis, cognitive impairment or dementia that could interfere with questionnaire completion, another active malignancy, and age younger than 40 or older than 80 years.

For data collection, the following instruments were used: a sociodemographic questionnaire specifically designed for this research, the Symptom Checklist-90-R (SCL-90-R), the Connor–Davidson Resilience Scale 25 (CD-RISC-25) and the World Health Organization Quality of Life Scale (WHOQOL-BREF).

The study-specific sociodemographic questionnaire was used for descriptive and covariate information rather than as a psychometric scale. It recorded sex, age, place of residence (urban or rural), self-reported economic status (above average, average, or low), education, marital and employment status, smoking, alcohol consumption, and medical history.

The Symptom Checklist-90-R (SCL-90-R) contains 90 self-report items rated from 0 (not at all) to 4 (extremely) and assesses nine symptom dimensions: somatization, obsessive-compulsive symptoms, interpersonal sensitivity, depression, anxiety, hostility, phobic anxiety, paranoid ideation, and psychoticism. Dimension scores were calculated as the mean of completed items assigned to each dimension. The Global Severity Index (GSI) was calculated as the sum of all item scores divided by the number of completed items; the Positive Symptom Total (PST) as the number of items scored above 0; and the Positive Symptom Distress Index (PSDI) as the sum of item scores divided by the PST. Higher scores indicate greater psychological symptom burden [24].

The 25-item Connor–Davidson Resilience Scale (CD-RISC-25) uses a five-point response scale from 0 (not true at all) to 4 (true nearly all the time). Item scores are summed to give a total score ranging from 0 to 100, with higher scores indicating greater resilience [25].

The World Health Organization Quality of Life-BREF (WHOQOL-BREF) contains 26 items rated on five-point scales. Following the standard scoring procedure, domain scores for physical health, psychological health, social relationships, and environment were transformed to a 0–100 scale, with higher scores indicating better quality of life. No overall WHOQOL-BREF score was calculated [26].

Religious involvement was assessed using the five-item Duke University Religion Index (DUREL), which yields three separate components: organizational religious activity (one item; score range of 1–6), non-organizational religious activity (one item; score range of 1–6), and intrinsic religiosity (three items; score range of 3–15). The components were analyzed separately and were not combined into a total score. In the original coding used in this study, lower scores indicate greater religious involvement [27].

All questionnaires used were standardized, validated, and approved by their respective authors for use in this research. Before completing the questionnaires, participants were informed about the purpose and objectives of the study and were assured of complete anonymity. Questionnaires were self-administered.

The study was approved by the Ethics Committee of University Clinical Hospital Mostar.

The analyzed sample comprised all consenting participants who met the eligibility criteria during the study period; consequently, the study may have had limited power to detect small group differences.

### Statistical Analysis

Statistical analyses were performed using IBM SPSS Statistics, version 25 (IBM Corp., Armonk, NY, USA). Data completeness, distributions, and outliers were examined before analysis. Normality was assessed using histograms, Q-Q plots, and the Shapiro–Wilk test. Categorical variables are reported as *n* (%), and continuous variables as mean +/− standard deviation. Between-group differences were assessed using independent-samples Student’s *t*-tests for continuous variables and chi-square or Fisher’s exact tests for categorical variables, as appropriate. Spearman’s rank-correlation coefficient was used to evaluate associations among SCL-90-R scores, CD-RISC, DUREL components, and WHOQOL-BREF domains. A MANCOVA examined the effect of group (recurrent NMSC vs. treated once) on six dependent variables: GSI, the four WHOQOL-BREF domain scores, and CD-RISC. The model was adjusted for age, sex, and economic status, selected as clinically relevant potential confounders. Because Box’s test indicated inequality of covariance matrices, Pillai’s Trace was used as the primary multivariate statistic. Estimated marginal means with 95% confidence intervals and partial eta squared are reported. Follow-up univariate *p* values were evaluated using a Bonferroni-adjusted significance threshold of 0.0083 (0.05/6); unadjusted *p* values are shown for transparency. Pairwise deletion was used for correlations when individual DUREL or PSDI values were unavailable. All other tests were two-sided.

## 3. Results

Comparison of sociodemographic and clinical characteristics between patients (Table 1) who underwent surgery for NMSC only once and patients with recurrent NMSC did not show statistically significant differences in most of the observed variables. A statistically significant difference between the groups was found only for the variable of previous surgery.

Analysis of age differences between patients in the test and control group showed no statistically significant difference. The average age of patients treated for NMSC only once was 67.59 ± 6.13 years, while the group of patients treated for recurrent NMSC was 66.32 ± 7.21 years. The results of the *t*-test showed that the difference between the groups was not statistically significant, indicating that the groups were comparable with respect to age.

Patients with recurrent NMSC had higher scores across all nine SCL-90-R symptom dimensions than patients treated once (Table 2). They also had higher GSI, PST, and PSDI scores (all *p* < 0.001), indicating greater overall symptom severity, a greater number of reported symptoms, and greater average distress among endorsed symptoms. In the unadjusted comparison, the recurrent NMSC group had a higher mean CD-RISC score (*p* = 0.021). Physical health, psychological health, and social relationships scores were lower in the recurrent group, whereas the environmental domain and all three DUREL components did not differ significantly between groups.

The number of participants varied for DUREL components because of missing or out-of-range responses: organizational religious activity, *n* = 157; non-organizational religious activity, *n* = 160; and intrinsic religiosity, *n* = 154. Values are presented as mean ± standard deviation; absolute t values are reported. GSI, Global Severity Index; PST, Positive Symptom Total; PSDI, Positive Symptom Distress Index; CD-RISC, Connor–Davidson Resilience Scale; WHOQOL-BREF, World Health Organization Quality of Life-BREF; DUREL, Duke University Religion Index.

Patients with recurrent non-melanoma skin cancer had significantly higher scores across all nine SCL-90-R symptom dimensions compared with patients who had undergone surgery only once (Table 2). Significant group differences were also observed for all three global SCL-90-R indices, with recurrent cases showing greater overall symptom severity, a higher number of positive symptoms, and greater average distress associated with reported symptoms (all *p* < 0.001).

Patients with recurrent NMSC had a significantly higher mean CD-RISC score than patients who had undergone surgery only once, indicating higher levels of resilience in the recurrent NMSC group (*p* = 0.021). Regarding quality of life, patients with recurrent NMSC had significantly lower scores in physical health (*p* = 0.049), psychological health (*p* < 0.001), and social relationships domains (*p* = 0.032). No statistically significant difference was found in the environmental domain (*p* = 0.225).

No statistically significant group differences were found in organizational religious activity (*p* = 0.188), non-organizational religious activity (*p* = 0.581), or intrinsic religiosity (*p* = 0.194), as measured by the DUREL.

Spearman’s correlation analysis showed that all nine SCL-90-R symptom dimensions were significantly negatively associated with all four WHOQOL-BREF domains (Table 3). The correlations were generally weak to moderate, ranging from ρ = −0.165 to ρ = −0.463. The strongest associations were observed between interpersonal sensitivity and psychological health (ρ = −0.463), phobic anxiety and physical health (ρ = −0.462), and interpersonal sensitivity and physical health (ρ = −0.461; all *p* < 0.001). These findings indicate that greater psychological symptom severity was consistently associated with poorer quality of life.

The three global SCL-90-R indices (Table 3) were also significantly negatively associated with all quality-of-life domains. Higher GSI, PST, and PSDI scores were related to poorer physical health, psychological health, social relationships, and environmental quality of life (all *p* ≤ 0.008). The strongest associations were observed for GSI with physical health (ρ = −0.462), GSI with psychological health (ρ = −0.457), and PST with psychological health (ρ = −0.445).

CD-RISC scores were not significantly associated with any WHOQOL-BREF domain (all *p* > 0.05). Given that lower DUREL scores indicate greater religiosity, greater organizational and non-organizational religious activity was associated with better quality of life in the social relationships domain. Greater intrinsic religiosity was associated with better physical health, social relationships, and environmental quality of life, whereas its association with psychological health was not statistically significant.

A multivariate analysis of covariance (Table 4) was conducted to examine group differences in psychological symptom severity, quality-of-life domains, and resilience while adjusting for age, sex, and economic status. Because Box’s test indicated inequality of covariance matrices, Pillai’s Trace was used as the primary multivariate statistic. The overall multivariate effect of group was statistically significant, Pillai’s Trace = 0.235, *F*(6,149) = 7.641, *p* < 0.001, and partial η^2^ = 0.235.

Follow-up univariate analyses showed that patients with recurrent NMSC had significantly higher adjusted GSI scores than patients who had undergone surgery only once, *F*(1.154) = 21.433, *p* < 0.001, partial η^2^ = 0.122. Patients with recurrent NMSC also had significantly lower adjusted psychological health scores, *F*(1.154) = 14.732, *p* < 0.001, and partial η^2^ = 0.087. A nominal difference was observed in the social relationships domain, *F*(1.154) = 4.311, unadjusted *p* = 0.040, partial η^2^ = 0.027; however, this association did not remain significant after correction for multiple follow-up tests.

No statistically significant adjusted group differences were found for physical health (*p* = 0.067), the environmental domain (*p* = 0.368), or resilience measured by the CD-RISC (*p* = 0.097).

## 4. Discussion

The results of this study indicate a complex interrelationship of psychological, sociodemographic and clinical factors in patients operated for non-melanoma skin cancer, with a special emphasis on patients with recurrent disease.

Most measured sociodemographic characteristics and age did not differ significantly between groups. However, absence of statistically significant differences does not establish complete comparability, and unmeasured clinical factors may have differed. The analyses therefore adjusted for age, sex, and economic status, but residual confounding cannot be excluded.

Patients with recurrent NMSC reported higher scores across all nine SCL-90-R symptom dimensions and all three global indices. In unadjusted comparisons, they also had lower physical, psychological, and social WHOQOL-BREF domain scores. In the adjusted multivariate analysis, recurrent disease status remained associated with higher GSI and lower psychological health after correction for multiple follow-up tests. The nominal difference in social relationships did not remain significant after Bonferroni correction, and adjusted differences in physical health and environment were not significant. These cross-sectional findings identify an association between recurrence status and psychological burden. They are broadly consistent with the literature describing fear of cancer recurrence as an important concern among cancer survivors [28,29,30,31,32,33,34].

Greater psychological symptom severity was consistently associated with poorer quality of life across all four WHOQOL-BREF domains. By contrast, CD-RISC was not significantly correlated with any WHOQOL-BREF domain. Although the recurrent group had a higher unadjusted mean CD-RISC score, the adjusted group difference was not statistically significant. Resilience should therefore not be interpreted as either lower in recurrent cases or as a demonstrated protective factor in this dataset. This differs from studies reporting associations among resilience, distress, and quality of life in other cancer populations [19,20,35,36], possibly reflecting differences in diagnosis, timing, clinical context, or measurement.

Among the adjusted follow-up outcomes, GSI showed the largest group effect, followed by psychological health. The social relationships result was nominally significant at the conventional 0.05 level but did not meet the Bonferroni-adjusted threshold. No adjusted group differences were found for physical health, environment, or CD-RISC. Accordingly, the most robust findings concern overall psychological symptom severity and psychological quality of life.

### 4.1. Limitations

Several limitations should be considered. First, the cross-sectional design and absence of preoperative or pre-recurrence measurements prevent conclusions about temporal sequence or causality. Second, outcomes were based on self-report questionnaires and may be affected by response and recall bias. Third, the single-center sample, unequal group sizes, absence of individual matching, and exclusion of patients with previous psychiatric diagnoses limit generalizability. No formal a priori sample-size calculation was performed, which may have limited detection of small effects.

Although tumors were restricted by location and size, potentially relevant clinical factors such as exact defect and scar characteristics, number of previous procedures, time since the preceding operation, histological subtype, reconstructive complexity, and previous radiotherapy were not fully modeled. Residual confounding is therefore possible. Finally, multiple outcomes and exploratory correlations were examined; the principal adjusted findings were interpreted using correction for six univariate follow-up tests.

Future multicenter longitudinal studies should include baseline and repeated assessments, predefined primary outcomes, formal sample-size calculations, and more detailed clinical covariates to clarify changes in psychological symptoms over time.

### 4.2. Clinical Implications

The findings support incorporating brief psychological screening into follow-up care for patients with recurrent NMSC. Patients with elevated distress should receive further assessment and, when indicated, referral for psychological or psychiatric support. Longitudinal evaluation is needed to determine whether such pathways improve quality of life or treatment outcomes.

## 5. Conclusions

Recurrent facial NMSC status was associated with higher overall psychological symptom severity and lower psychological quality of life after adjustment for age, sex, and economic status and correction for multiple follow-up tests. The adjusted difference in resilience was not statistically significant. These findings support psychological screening as part of multidisciplinary follow-up while highlighting the need for longitudinal research before causal conclusions can be drawn.

## Figures and Tables

**Table 1:** Sociodemographic and clinical data by group.

	Group					
	Control Group		Test Group			
	N	%	N	%	χ^2^	*p*
Sex					0.600	0.439
F	34	54.0	45	46.4		
M	29	46.0	52	53.6		
Level of education					6.806	0.124 *
No degree	1	1.6	1	1.0		
8th/10th grade	8	12.7	28	28.9		
High school	32	50.8	36	37.1		
College	10	15.9	13	13.4		
University	12	19.0	19	19.6		
Marital status					1.289	0.798 *
Single	5	7.9	13	13.4		
Married	43	68.3	62	63.9		
Divorced	1	1.6	2	2.1		
Widowed	14	22.2	20	20.6		
Work status					3.038	0.290
Employed	5	7.9	8	8.2		
Unemployed	5	7.9	17	17.5		
Retired	53	84.1	72	74.2		
Economic status					2.641	0.267
Above average	4	6.3	5	5.2		
Average	56	88.9	80	82.5		
Low	3	4.8	12	12.4		
Living place					0.019	0.890
City/town	29	46.0	47	48.5		
Country	34	54.0	50	51.5		
Smoking					2.972	0.400 *
Non smoker	50	79.4	81	83.5		
Up to 10	4	6.3	3	3.1		
Up to 20	8	12.7	8	8.2		
Up to 40	1	1.6	5	5.2		
Alcohol					1.291	0.885 *
No	33	52.4	55	56.7		
On occasion	22	34.9	34	35.1		
On weekends	3	4.8	3	3.1		
Often	2	3.2	2	2.1		
Daily	3	4.8	3	3.1		
Family medical history					2.849	0.860 *
Diabetes	6	9.5	9	9.3		
Hypertension	6	9.5	12	12.4		
Heart diseases	3	4.8	9	9.3		
Kidney diseases	2	3.2	4	4.1		
Neurologic diseases	2	3.2	1	1.0		
Thyroid gland	3	4.8	6	6.2		
Other	41	65.1	56	57.7		
Personal medical history					2.891	0.226 *
Diabetes	1	1.6	2	2.1		
Hypertension	2	3.2	10	10.3		
Other	60	95.2	85	87.6		
Previous diseases	50	79.4	93	95.9	9.295	0.002

* Fisher’s Exact Test.

**Table 2:** Comparison of psychological symptoms, resilience, quality of life, and religiosity between patients treated once and patients with recurrent non-melanoma skin cancer.

Variable	Control Group (*n* = 63)	Recurrent NMSC Group (*n* = 97)	*t*	*p*
SCL-90-R				
Somatization	0.73 ± 0.51	1.01 ± 0.56	3.17	0.002
Obsessive-compulsive symptoms	0.77 ± 0.54	1.04 ± 0.56	2.96	0.004
Interpersonal sensitivity	0.62 ± 0.49	0.98 ± 0.57	4.11	<0.001
Depression	0.68 ± 0.43	1.04 ± 0.50	4.73	<0.001
Anxiety	0.65 ± 0.46	1.01 ± 0.54	4.36	<0.001
Hostility	0.36 ± 0.37	0.83 ± 0.63	5.35	<0.001
Phobic anxiety	0.45 ± 0.46	0.82 ± 0.59	4.17	<0.001
Paranoid ideation	0.57 ± 0.41	0.94 ± 0.59	4.31	<0.001
Psychoticism	0.51 ± 0.47	0.90 ± 0.56	4.62	<0.001
GSI	0.62 ± 0.42	0.97 ± 0.51	4.66	<0.001
PST	39.70 ± 21.92	56.60 ± 23.81	4.50	<0.001
PSDI	1.33 ± 0.26	1.50 ± 0.30	3.65	<0.001
Resilience				
CD-RISC	49.73 ± 17.68	56.12 ± 16.50	2.33	0.021
WHOQOL-BREF				
Physical health	64.29 ± 11.76	60.01 ± 14.21	1.99	0.049
Psychological health	63.43 ± 10.08	56.23 ± 11.40	4.08	<0.001
Social relationships	75.53 ± 14.66	70.27 ± 15.26	2.16	0.032
Environment	64.48 ± 11.20	62.27 ± 11.23	1.22	0.225
DUREL				
Organizational religious activity	3.21 ± 1.33	3.51 ± 1.40	1.322	0.188
Non-organizational religious activity	2.87 ± 1.75	3.03 ± 1.78	0.553	0.581
Intrinsic religiosity	6.15 ± 2.92	6.85 ± 3.47	1.304	0.194

**Table 3:** Correlations of psychological symptoms, resilience, and religiosity with quality-of-life domains.

Variable	Physical Health	Psychological Health	Social Relationships	Environment
SCL-90-R				
Somatization	−0.382 **	−0.372 **	−0.174 *	−0.318 **
Obsessive-compulsive symptoms	−0.454 **	−0.415 **	−0.181 *	−0.380 **
Interpersonal sensitivity	−0.461 **	−0.463 **	−0.259 **	−0.321 **
Depression	−0.394 **	−0.420 **	−0.232 **	−0.360 **
Anxiety	−0.435 **	−0.433 **	−0.189 *	−0.396 **
Hostility	−0.340 **	−0.390 **	−0.244 **	−0.347 **
Phobic anxiety	−0.462 **	−0.377 **	−0.165 *	−0.366 **
Paranoid ideation	−0.405 **	−0.370 **	−0.241 **	−0.382 **
Psychoticism	−0.355 **	−0.425 **	−0.187 *	−0.384 **
GSI	−0.462 **	−0.457 **	−0.235 **	−0.400 **
PST	−0.433 **	−0.445 **	−0.231 **	−0.404 **
PSDI	−0.367 **	−0.397 **	−0.226 **	−0.210 **
Resilience				
CD-RISC	0.034	0.082	0.036	0.126
DUREL				
Organizational religious activity	−0.010	−0.067	−0.269 **	0.030
Non-organizational religious activity	−0.091	0.066	−0.175 *	−0.041
Intrinsic religiosity	−0.168 *	−0.139	−0.224 **	−0.240 **

Values are Spearman’s correlation coefficients (ρ). * *p* < 0.05; ** *p* < 0.01. Pairwise sample sizes ranged from 153 to 160 because of missing DUREL values and one undefined PSDI score. GSI, Global Severity Index; PST, Positive Symptom Total; PSDI, Positive Symptom Distress Index; CD-RISC, Connor–Davidson Resilience Scale; DUREL, Duke University Religion Index. Lower DUREL scores indicate greater organizational religious activity, non-organizational religious activity, and intrinsic religiosity.

**Table 4:** Adjusted differences in psychological symptoms, quality of life, and resilience between study groups.

Outcome	Treated Once, Adj. Mean (95% CI)	Recurrent NMSC, Adj. Mean (95% CI)	*F*	*p*	Partial Eta Squared
GSI	0.666 (0.499–0.834)	1.028 (0.881–1.175)	21.433	<0.001	0.122
Physical health	60.952 (56.280–65.624)	56.941 (52.854–61.027)	3.398	0.067	0.022
Psychological health	60.193 (56.395–63.992)	53.403 (50.081–56.725)	14.732	<0.001	0.087
Social relationships	69.623 (64.467–74.778)	64.637 (60.129–69.146)	4.311	0.040	0.027
Environment	60.392 (56.544–64.241)	58.775 (55.409–62.141)	0.815	0.368	0.005
CD-RISC	50.381 (44.765–55.997)	54.743 (49.832–59.654)	2.782	0.097	0.018

Values are estimated marginal means with 95% confidence intervals, adjusted for age, sex, and economic status. The overall multivariate group effect was significant: Pillai’s Trace = 0.235, *F*(6.149) = 7.641, *p* < 0.001, partial η^2^ = 0.235. GSI, Global Severity Index; CD-RISC, Connor–Davidson Resilience Scale; NMSC, non-melanoma skin cancer. All univariate tests had 1 and 154 degrees of freedom. Univariate *p* values are unadjusted; after Bonferroni correction for six follow-up tests (α = 0.0083), only GSI and psychological health remained statistically significant.

## Data Availability

The data presented in this study are available on request from the corresponding author due to ethical reasons.
